# No frugal innovation without frugal evaluation: the Global IDEAL Sub-Framework

**DOI:** 10.1136/bmjsit-2023-000248

**Published:** 2024-06-12

**Authors:** William S Bolton, Noel K Aruparayil, Bonnie Cundill, Peter McCulloch, Jesudian Gnanaraj, Ibrahim Bundu, Peter R Culmer, Julia M Brown, Julian Scott, David G Jayne

**Affiliations:** 1 Leeds Institute of Medical Research, University of Leeds, School of Medicine, Leeds, UK; 2 Leeds Institute of Clinical Trials Research, University of Leeds, School of Medicine, Leeds, UK; 3 Nuffield Department of Surgical Science, University of Oxford, Oxford, UK; 4 Rural Surgery Innovations, Dimapur, Nagaland, India; 5 Rural Surgery Innovations Private Limited, Karunya Institute of Technology and Sciences, Coimbatore, India; 6 Connaught Hospital, Freetown, Sierra Leone; 7 School of Mechanical Engineering, University of Leeds, Leeds, UK

**Keywords:** Device Evaluation

## Abstract

**Objective:**

The Global IDEAL Sub-Framework Study aimed to combine the intended effects of the 2009/2019 IDEAL (Idea, Development, Exploration, Assessment, Long-term study) Framework recommendations on evaluating surgical innovation with the vision outlined by the 2015 Lancet Commission on Global Surgery to provide recommendations for evaluating surgical innovation in low-resource environments.

**Design:**

A mixture of methods including an online global survey and semistructured interviews (SSIs). Quantitative data were summarized with descriptive statistics and qualitative data were analyzed using the Framework Method.

**Participants:**

Surgeons and surgical researchers from any country.

**Main outcome measures:**

Findings were used to suggest the nature of adaptations to the IDEAL Framework to address the particular problems of evaluation in low-resource settings.

**Results:**

The online survey yielded 66 responses representing experience from 40 countries, and nine individual SSIs were conducted. Most respondents (n=49; 74.2%) had experience evaluating surgical technologies across a range of life cycle stages. Innovation was most frequently adopted based on colleague recommendation or clinical evaluation in other countries. Four themes emerged, centered around: frugal innovation in technological development; evaluating the same technology/innovation in different contexts; additional methodologies important in evaluation of surgical innovation in low/middle-income countries; and support for low-income country researchers along the evaluation pathway.

**Conclusions:**

The Global IDEAL Sub-Framework provides suggestions for modified IDEAL recommendations aimed at dealing with the special problems found in this setting. These will require validation in a stakeholder consensus forum, and qualitative assessment in pilot studies. From assisting researchers with identification of the correct evaluation stage, to providing context-specific recommendations relevant to the whole evaluation pathway, this process will aim to develop a comprehensive and applicable set of guidance that will benefit surgical innovation and patients globally.

**WHAT IS ALREADY KNOWN ON THIS TOPIC**

- Evaluating surgical innovation is challenging and the IDEAL (Idea, Development, Exploration, Assessment, Long-term study) Framework has been guiding surgical researchers for over a decade. Its applicability and relevance to surgeons working in low/middle-income countries (LMICs) were not clear, but the challenges in these settings are different in specific ways and more pronounced.

**WHAT THIS STUDY ADDS**

- Based on our findings, we suggest how the surgeons and researchers in LMICs might best modify the IDEAL guidance to assist them in evaluating local surgical innovation.

**HOW THIS STUDY MIGHT AFFECT RESEARCH, PRACTICE OR POLICY**

- This is the first study to focus on developing context-specific guidance for surgical innovation evaluation in low-resource environments. More work is required but such guidance could be used with advantage by surgeons, researchers, and policymakers worldwide.

## Introduction

There persists a global lack of evaluation for surgical innovation.^1 2^ Methodological challenges associated with generating scientific evidence for surgical innovations include complexity and standardization of the intervention, especially when conducting the rigorous evaluations needed to persuade policymakers and surgeons to adopt.^2^ The IDEAL Framework is maintained by the IDEAL Collaboration which is a multidisciplinary group of researchers, methodologists, and clinicians (https://www.ideal-collaboration.net). The IDEAL Framework was first published in 2009, and updated in 2019, to address these challenges. It provides structured recommendations outlining the systematic evaluation of surgical innovation based on the stage of evolution of the procedure or technology.^2 3^ IDEAL (Idea, Development, Exploration, Assessment, Long-term study) consists of five stages. Stage 1 focuses on ‘first-in-human’ studies, involving a small number of participants, where the main outcomes are proof of concept. Stage 2 is split into 2a and 2b. Stage 2a includes a small number of selected participants in a single group design, aiming to document the evolution of the procedure or technology towards a stable, optimized version. Stage 2b builds on this using collaborative prospective cohort studies and feasibility randomized controlled trials (RCTs) to generate clinical consensus around indications, overall effectiveness, and quality of delivery, focusing on feasibility and short-term safety outcomes. Stage 3 seeks definitive comparative evidence of clinical and cost effectiveness, normally involving multicenter RCTs with longer follow-up. Finally, stage 4 takes the form of long-term surveillance studies such as registries or routine databases. Here, the focus is on ensuring and maintaining standards. In 2016, the IDEAL-D Framework modified the IDEAL recommendations to include considerations specifically for medical devices, including a new IDEAL stage 0 for preclinical development.^4^ In 2018, a bibliometric analysis investigated the uptake and use of the IDEAL Framework for conducting stage 1, 2a and 2b studies, and appraised 38 publications that used the framework.^5^ The findings demonstrated an upward trend in adoption of the framework during the evaluation of surgical innovation at this earlier stage but noted that that only one study had a first author from a low/middle-income country (LMIC). This suggests that use of the framework is increasing (though not widespread) and implies that more dissemination and education work may be needed. The results from this also suggest that uptake of IDEAL in LMICs is lacking, which might signal a lack of applicability to these contexts in its current form.

In 2015, the Lancet Commission on Global Surgery created a targeted roadmap to upscale global surgical care by 2030.^6^ Key enabling factors to achieve these targets include the development and deployment of technologies. Many of the challenges faced in designing and evaluating surgical technologies are even more pronounced in the low-resource environments^7^ found in LMICs, although resource constraints also affect healthcare systems in high-income countries (HICs) to a differing extent.^8^

The original IDEAL Framework did not focus on evaluating technologies for low-resource environments, which present a unique set of challenges which may require modification of the recommendations. Expanding the IDEAL Framework to increase its relevance and utility for low-resource contexts will facilitate the design, evaluation, translation and adoption of surgical innovation globally. There are specific challenges with evaluation in LMICs: first, there is uncertainty around when re-evaluation is needed for interventions with level one evidence in HICs, and why this might be the case; second, there are special knowledge, capacity and resource constraints in LMIC settings and how these can best be addressed remains unclear. Therefore, the Global IDEAL Sub-Framework Study defined the unique barriers and facilitating strategies along the evaluation pathway of surgical innovation in LMICs, and developed adaptations to the original IDEAL Framework to help evidence generation and support adoption. Our aim was to combine the intended effects of the IDEAL Framework and the Lancet Commission on Global Surgery to provide a comprehensive practical framework for surgical innovation in LMICs.

## Methods

A mixed-methods study was conducted combining a semiquantitative online survey and key informant semistructured interviews (SSIs). Barriers and facilitators in evaluating surgical innovation described by survey respondents were used to frame themes for further exploration through SSIs. English-speaking surgeons or researchers involved in surgical care or surgical innovation in LMICs were recruited. For the purposes of this study, LMIC was defined as any country appearing on the Development Assistance Committee list of Official Development Assistance recipients. The overall process was as follows: online survey was designed and piloted, which was disseminated digitally via the internet; then, respondents of the survey were invited to take part in a further SSI if they wished to be more involved; these were conducted using virtual teleconferencing technologies.

### Online survey design and execution

A cross-sectional survey using a self-reported, anonymized, online questionnaire was designed in English and built in Jisc Online Surveys (Bristol, UK). The content covered demographics including work setting, experience of surgical technology evaluation, and perceived barriers or facilitating factors to conducting each IDEAL stage evaluation in their context. Participants were encouraged to propose alternatives to existing proposals for study designs and facilitating strategies recommended in existing evaluation pathway. Free-text boxes allowed respondents the opportunity to expand their answers. The final survey design is available in online supplemental appendix 2.

10.1136/bmjsit-2023-000248.supp2 Supplementary data

To provide a generalizable and representative global evidence base, the survey was distributed using snowball sampling through relevant collaborative groups in a range of HICs and LMICs. Key mailing lists and membership groups were approached for dissemination, including GlobalSurg Collaborative, G4 Alliance, Association of Rural Surgeons of India, West African College of Surgeons, and the College of Surgeons of East, Central and Southern Africa. Social media platforms were used to expand reach to a broader audience. Data were collected over a 12-week period in 2019.

### Participant involvement

The survey was piloted with surgeons from Sierra Leone to ensure face and content validity. During the pilot, discussions on the design and content of the survey identified missing or surplus topics, and ensured the phrasing and flow of the survey was optimized before dissemination. Patients were not involved at this stage.

### Semistructured interviews

Respondents to the online questionnaire were invited to take part in the interviews. Interviews were conducted either face-to-face or using video teleconference platforms such as Zoom (Zoom Corporation, San Jose, USA) and WhatsApp (WhatsApp, California, USA). All interviews were in English and conducted by a researcher trained in qualitative methods. Interviews were audio recorded and transcribed verbatim. Interviews were semistructured, using a topic guide to ensure core topics were covered, using prompts and follow-up questions based on responses. A copy of the topic guide is available in online supplemental appendix 2.

### Data analysis

Descriptive statistics summarized demographics and categorical or Likert scale responses, and tabulation was performed on Microsoft Excel V.16.50. Free-text box responses were thematically analyzed and included in the overall qualitative data analysis.

Qualitative data analysis was an ongoing, iterative process. Preliminary analysis began during data collection. This involved conducting a reflective debrief after each interview. Thematic analysis using Framework Methodology was used to analyze the complete data and inductively identify themes.^9^ This approach was chosen as it identifies commonalities and differences in qualitative data before focusing on relationships within the data, leading to descriptive or explanatory conclusions clustered around themes. The wider research team then assessed emerging themes, contributing to iterative refinement and interpretation of the results. Qualitative data presentation is constructed in line with the Standards for Reporting Qualitative Research checklist for qualitative studies.^10^

### Framework components and derivation

Proposals for recommendations within the Global IDEAL Sub-Framework were derived by mapping the barriers and facilitators identified in interviews to the corresponding original IDEAL Framework recommendations. Additional methodologies or new framework components identified from the survey or interviews were then added. These approaches have been used to help bridge the theory–research–practice divide in global and public health initiatives.^11 12^

This initial iteration of the Global Surgical Innovation IDEAL Sub-Framework was presented at a workshop during the IDEAL Virtual Congress, April 15–16, 2021, bringing together clinicians and researchers from all over the world including colleagues from LMICs. Additional modifications and suggestions were discussed and incorporated into the Global IDEAL Sub-Framework that is presented in this paper.

## Results

### Online survey and interviews

#### Participants

**Figure 1:**
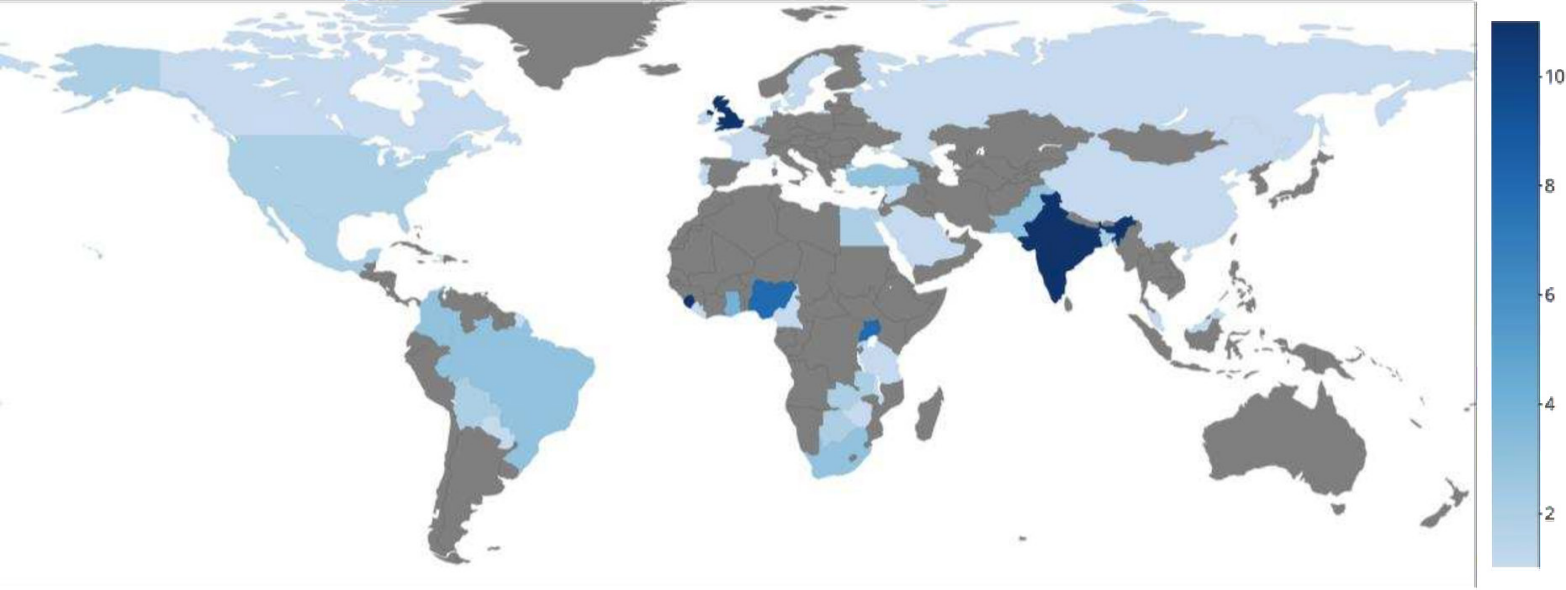
Survey participant location in the global map (n=number of participants from that country).

The online survey yielded 66 responses representing experience from 40 countries. 28 (42.4%) were consultant surgeons/attending physicians and 23 (34.8%) trainees/resident surgeons. Nine (13.6%) were researcher/academic/trialist/methodologist. The remaining respondents were obstetrics & gynecology (n=5; 7.6%), surgical associate/surgical officer (n=2; 3%) and anesthetist/anesthesiologist (n=2; 3%). Four (6.1%) recorded ‘other’. Three-quarters of respondents were male (50; 75.8%). The majority (n=46; 69.7%) worked in public hospitals in urban settings (n=56; 84.8%). The respondent demographics are summarized in table 1 and figure 1. Nine respondents were included in the interview stage after expressing interest.

**Table 1:** Respondent demographics and places of work

Professional experience		Hospitals currently working with	
Consultant surgeon/attending physician	28	**No of beds**	
Trainee/resident surgeon	23	Less than 50	6
Researcher/academic/trialist/methodologist	9	50–99	7
Obstetrician/gynecologist	5	100–199	7
Surgical associate/surgical officer	2	200–499	15
Anesthetist	2	500–999	17
Other	4	1000+	14
**Surgical specialty experience**		**Type of hospital**	
General	41	Public	46
Trauma & orthopedics	11	Private	7
Obstetrics & gynecology	10	Mixed public and private	8
Pediatric	7	NGO/charity	5
Neurological	6	**Area served**	
Urological	5	Urban	56
ENT	4	Rural	10
Plastic and reconstructive	2	**Countries currently working in**	
Vascular	2	India (11), Sierra Leone (11), UK (11), Nigeria (8), Uganda (8), Ghana (4), Brazil (3), Colombia (3), Pakistan (3), South Africa (3), Turkey (3), Bangladesh (2), Bolivia (2), Botswana (2), Egypt (2), Ethiopia (2), Mexico (2), Netherlands (2), Rwanda (2), USA (2), Zambia (2), Cameroon (1), Canada (1), China (1), Denmark (1), France (1), Haiti (1), Ireland (1), Liberia (1), Malawi (1), Malaysia (1), Paraguay (1), Portugal (1), Russia (1), Saudi Arabia (1), Sweden (1), Syria (1), Tanzania (1), West Bank and Gaza (1), Zimbabwe (1)	
Anesthetics	2		
Ophthalmology	1		
Cardiac	0		
Other	3		

ENT, ear, nose, and throat; NGO, non-governmental organization.

#### Experience of evaluating innovation

**Figure 2:**
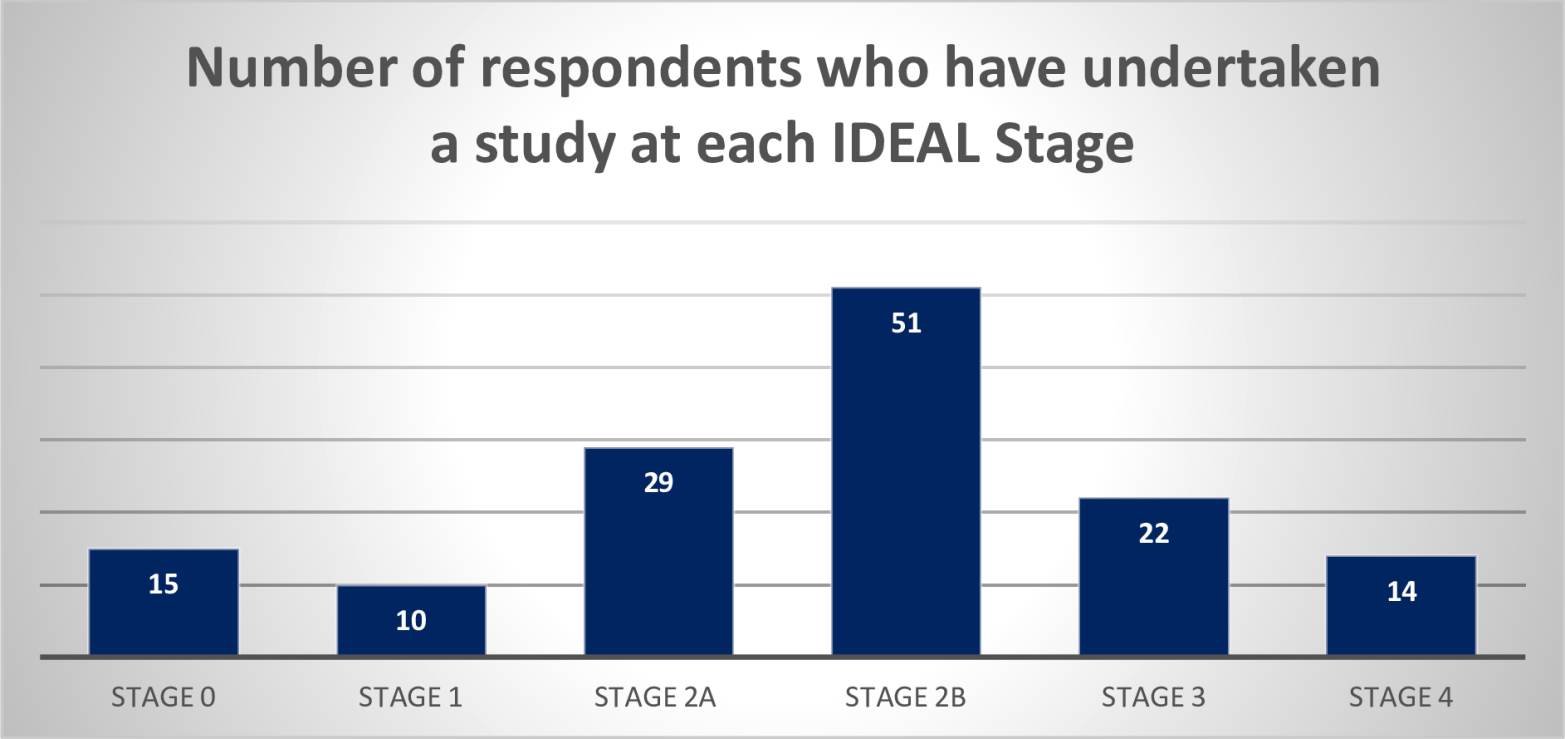
The number of respondents who have undertaken a study at each IDEAL stage (short descriptors were used to describe the IDEAL stages). IDEAL, Idea, Development, Exploration, Assessment, Long-term study.

The majority (n=48; 72.7%) had experience in clinical research with patients, and the remaining either had experience with preclinical research (n=15; 22.7%) or had no prior experience (n=15; 22.7%) (respondents could select more than one option). The spread of evaluation experience mapped against IDEAL stages is shown in figure 2, with the majority having experience in IDEAL stage 2 studies. The conduct of IDEAL stage 1 studies was relatively uncommon. The majority (n=48; 72.8%) of participants felt that conducting this study design in LMICs was realistic in their experience. Most participants felt that conducting IDEAL stage 2a and 2b studies was realistic in their experience (n=51; 77.3% and n=44; 66.7%, respectively). For IDEAL stage 3 studies, over half (n=35; 53%) felt this design was realistic in their setting. Finally, 48 (72.7%) of participants felt IDEAL stage 4 studies were realistic in their setting.

**Figure 3:**
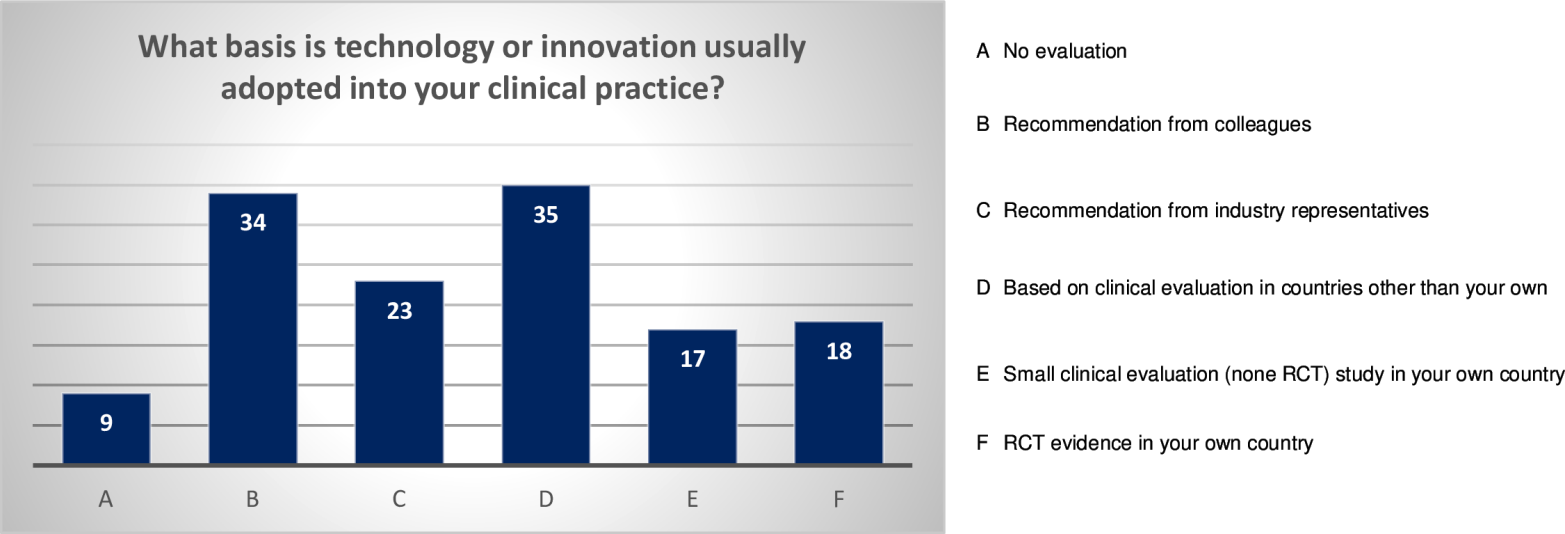
The basis of current technology or innovation adoption in the respondent’s experience. RCT, randomized controlled trial.

**Figure 4:**
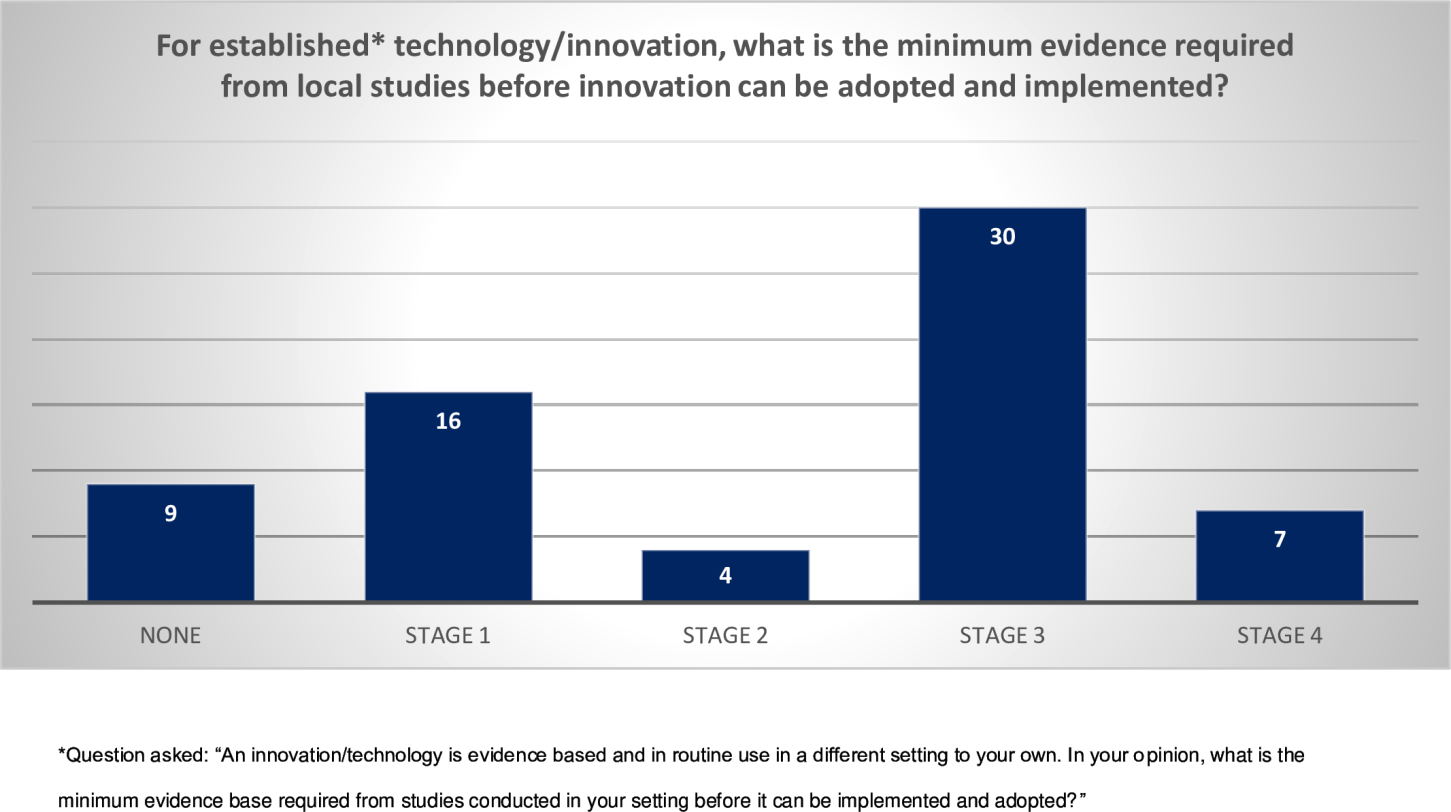
Respondents’ perceptions around evidence required from local studies before adopting a technology/innovation in established use in a context other than their own. Stage 0 refers to preclinical studies; stage 1 focuses on a small number of participants; stage 2 is split into 2a and 2b. Stage 2a includes a small number of selected participants in a single group design, stage 2b builds on this using prospective cohort studies and feasibility randomized controlled trials. Stage 3 seeks definitive comparative evidence of clinical and cost effectiveness; stage 4 takes the form of long-term surveillance studies.

Most (n=49; 74.2%) had experience evaluating surgical technologies. Respondents reported that technologies and innovations were most frequently adopted either based on colleague recommendation or on clinical evaluation in countries other than their own (figure 3). Almost half (n=30; 45.4%) of respondents perceived the need for evidence from a locally conducted RCT before adopting a technology/innovation that is in established use in a context other than their own (figure 4).

### Qualitative interview findings

#### Themes emerging through exploration of barriers and facilitators in surgical innovation evaluation in LMICs

Barriers and facilitators were used to frame four key themes that were considered important when evaluating surgical innovation in LMICs.

- Theme 1: frugal innovation in device development
- Theme 2: evaluating the same technology/innovation in multiple contexts
- Theme 3: additional methodologies important in evaluation of surgical innovation in LMICs
- Theme 4: support for researchers along the evaluation pathway

##### Theme 1: frugal innovation in device development

Several respondents had experience working on surgical technologies at the IDEAL stage 0 phase (preclinical). A reason for engaging in device development arose from the barriers associated with donation of medical devices from HICs to LMICs. For example, it was quoted, ‘Most of the time these do not work and end up in a donation graveyard’. This is because these devices were not specifically designed for use in these contexts and a lack of consumables or training, or issues with maintenance and repair limit the device usability. Concepts pertaining to the principle of frugal innovation arose on multiple occasions. This frequently involved ensuring devices were ‘lower cost’ but also recognized that ‘the innovation itself must be context specific’ with the need for ‘adaptive technology’ specific for surgical care in LMICs, noting that the ‘cultural acceptance of intervention, viability and sustainability in developing countries’ is paramount.

##### Theme 2: evaluating established technology/innovation in multiple contexts

The original IDEAL Framework was created with the evaluation of completely novel interventions in mind and originally specifically excluded established or existing technologies. In global surgery, surgeons are often faced with the need to evaluate technology already established in one context (often HICs) and they need to decide how best to progress in the context they currently work in. ‘The effectiveness, or appropriateness, of an intervention is absolutely context specific’ if, for example, the ‘training or additional equipment or follow-up’ is not available. The intervention itself and the study design may both need to be adjusted to the contact in a ‘kind of pre-analysis to assess the intervention and context readiness’. A specific issue that was frequently discussed was the need to avoid having to repeat IDEAL stage 3 (definitive RCTs) in every possible context due to impracticalities and stifling innovation adoption: ‘It’s not about is A better than B, it’s about can we reproduce the safety results in this setting’. IDEAL 2-like studies were the most common types of studies conducted by participants in this study. This was often because, when evaluating a technology in a new context, they needed to make their own evaluation of the balance of risks and benefits in their context. Thus, they ‘first want to make sure the innovation is safe, then identify where the risks lie’ and sought to reproduce previous reports in terms of achieving ‘similar results without excess harm’ before deciding that the technology/innovation could be adopted.

##### Theme 3: additional methodologies important in evaluation of surgical innovation in LMICs

A frequently discussed topic was the need to incorporate additional methodologies into existing IDEAL stages to increase the relevance of the framework for surgical researchers in LMICs. There were calls to revise the PICO question (Population, Intervention, Comparison, and Outcome) —‘The research question, make sure it is relevant and specific to our needs’ and to study ‘why and how they (intervention) work in this area’ recognizing the need for qualitative methodologies to explore these topics in more detail. There was a frequent desire to ‘focus on the training and implementation of the new technology’ and methods from implementation science including iterative development evaluation cycles and mixed methodologies were frequently raised as examples. Recommendations for stage 2b studies already call for qualitative studies and pay attention to learning curves. Health economics evaluations were seen as very relevant both by survey participants and interviewees, especially in LMICs where there is a need to ‘think of it as a public health issue and identify local and low-cost solutions’.

##### Theme 4: support for researchers along the evaluation pathway

A frequently discussed issue was the effect of the ‘lack of background knowledge in the basics of research methodologies’ on evolution and innovation adoptions. One respondent simply summarised the main areas of support needed as ‘money, knowledge, time’, explained further as funding to deliver research, better understanding of evaluation methods needed and protected time or workforce support to deliver the studies. To help with this, ‘effective local and international collaboration is essential’ and ‘better training and understanding in innovation pathways’ need to be fostered. To achieve sustained growth in the capacity of LMIC surgeons and researchers to evaluate their own work will require the development of respectful partnerships with HIC colleagues, in which the leadership of the LMIC surgeons is acknowledged alongside their need for guidance and mentoring in scientific methodology.

### The Global Surgical Innovation IDEAL Sub-Framework

A summary comparison of the proposals for recommendations characterizing the original IDEAL Framework and the new Global IDEAL Sub-Framework at each stage of evaluation is presented in table 2. The proposed modified recommendations build upon (rather than replace) the original IDEAL and IDEAL-D Frameworks which can be freely accessed here^2 4^: https://www.ideal-collaboration.net. The new recommendations proposed are as follows:

**Table 2:** Brief comparison of the features characterizing the original IDEAL/IDEAL-D and Global Surgical Innovation IDEAL Sub-Framework recommendations at each stage

Original IDEAL/IDEAL-D stage of innovation	Global Surgical Innovation IDEAL Sub-Framework recommendations and considerations
**Pre-IDEAL** **Stage selection**	
Silent but recognized: theoretical exercise only	Conceptual decision-making aid provided to select appropriate stage to enter the pathway based on rapid appraisal of existing evidence. This is a light touch formal risk assessment exercise looking at context differences within existing literature (population, geography, healthcare system). We recommend this is done before moving on with any stage of evaluation.
**Stage 0 Pre-clinical study** **Frugal innovation and context-centered design**	
Calls for standards for publication/registration of preclinical data to be established	Calls for consideration of the context-specific processes, human factors, system, and regulatory issues and for employing principles of frugal innovation.
**Stage 1 First-in-human study** **No change, but is it needed for established innovation?**	
First-in-human studies with compulsory confidential reporting of all wholly new innovations	No change, but recognition that much innovation in global surgery is not first-in-human and if so, this stage may not need to be duplicated.
**Stage 2a and 2b Feasibility study** **Key diffusers of innovation**	
2a: small uncontrolled cohort studies, usually single center, with consecutive case reporting and explanation of innovation development. Focus on technical details and feasibility.	These study designs are particularly suitable for evaluating the feasibility and safety of established innovations in a new context. Focus on innovation development to fit this context and capturing any unexpected consequences/outcomes.
2b: explanatory or feasibility RCTs, usually smaller in scale, focusing on safety and feasibility outcomes. Can be efficacy trials.	
**Stage 3 Effectiveness study** **Beyond the traditional RCT**	
RCTs, ideally multicenter, appropriately statistically powered. Aim to assess clinical effectiveness of interventions.	Consider the use of cluster randomized and stepped-wedge designs where appropriate. As with stage 2 studies, include in-built qualitative process evaluations and consider phased/hybrid RCT-implementation evaluation designs.
**Stage 4 Long-term monitoring study** **Engage with mixed-methods registries sooner**	
Comprehensive registries and databases for recording rare events, long-term outcomes and challenges in use.	Registries should be employed as soon as possible, including in conjunction with earlier stages.
Pan-stage considerations Some considerations are recommended for each stage and form more large-scale guidance for implementing the innovation evaluation pathway globally: Emphasis on employing in-built, protocol-driven mixed-methods approaches at each stage Fostering innovation culture guided by frugal innovation principles. In-built health economics evaluations to help make decisions about appropriate adoption and choose between innovations. Loco-regional and international collaboration led by LMIC researchers with support and mentoring from HIC colleagues. Pan-stage leadership of LMIC researchers, surgeons and patients/public in design, evaluation and adoption of innovation. Supporting researchers via training, methodological support, securing funding and identifying dissemination and advocacy opportunities.	

HIC, high-income country; IDEAL, Idea, Development, Exploration, Assessment, Long-term study; LMIC, low/middle-income country; RCTs, randomized controlled trials.

#### Pre-IDEAL: stage selection

A new stage is suggested to help the innovator decide where to enter the evaluation pathway based on their assessment of the local context and existing evidence for a specific innovation. Surgeons working in LMICs are often aiming to evaluate established innovations and want to adopt them safely in their own context. Decisions on the context-relevant evidence required to achieve a balance between benefits of swift innovation adoption and risks of potential harm through underevaluation should be made in collaboration with regulators, innovators, surgeons, and patients themselves. Local clinical expertise, professional ethical guidance and the views of local policymakers and other stakeholders need to be considered and consensus sought on a way ahead for evaluation. The pre-IDEAL stage selection tool is a high-level decision-making aid provided to focus this exercise and is provided in online supplemental appendix 1. If the innovation is completely novel and first-in-man studies have not been conducted, then the researcher should begin with stage 0 and progress sequentially no matter what the context.

10.1136/bmjsit-2023-000248.supp1 Supplementary data

#### IDEAL stage 0: adopt principles of frugal innovation and context-centered design

Present in the IDEAL-D Framework, this stage is retained here with a shift in focus, calling upon the researcher to ensure that the principles of frugal innovation are adhered to during the initial design of innovation. Frugal innovation refers to the concept of doing better with less. By concentrating on user-centered design, focusing on minimum required core functionalities to achieve the primary technological aim, reducing cost and waste, frugal innovation can produce elegant, context-specific solutions to complex problems.^13 14^ We recommend researchers employ context-centered design principles, including considering the innovation’s acceptability to differing contexts and populations.

#### IDEAL stage 1: no change, but is it needed for established innovation?

The original IDEAL stage 1 is compulsory for all entirely novel innovations and all first-in-human studies should be internationally registered, and this sub-framework endorses this principle. However, if the first-in-human studies have already been completed, then at this stage, we recommend the need to return to stage selection. We note that it may be reasonable to progress without repeating stage 1 again. We expect that for most established innovation, supplementary stage 1 studies in new contexts/populations/systems may not be required.

#### IDEAL stage 2a and 2b: key diffusers of innovation

We recommend the use of these study designs for when established innovation is evaluated in a new context. Given the relative simplicity of these studies, they can facilitate rapid, cost-effective evaluations that can mitigate safety concerns efficiently. The focus here is on checking that results of new evaluations are comparable with existing data in other contexts, while capturing any unexpected consequences arising from new contexts. It may be reasonable that if the results are acceptable at this stage, then adoption with ongoing monitoring via a registry is acceptable without the need for IDEAL stage 3 studies. We recommend supplementing these stage 2a, followed where the context makes this feasible by 2b studies with in-built qualitative process evaluations to provide richer contextual information. The iterative evaluation cycles described in IDEAL stage 2a could be further strengthened by including phased or hybrid evaluation–implementation cycles seen in implementation science techniques.^15^ This could be represented as an IDEAL stage 2a or 2b study followed by implementation and monitoring via a registry (IDEAL stage 4 study) with an ongoing process evaluation.

#### IDEAL stage 3: beyond the traditional RCT

An IDEAL stage 3 study is required if no stage 3 study has been completed for a specific innovation in any context. In this unusual situation, the problem of context interpretation would be inverted, and HIC clinicians would need to decide whether further 2a/2b studies were needed in their context before accepting the validity of the RCT result for their patients. If stage 3 evidence already exists in a different context, stage 2a and 2b studies in a low-income country context would be ethically adequate evidence for implementation if they confirmed satisfactory feasibility and safety results.^16^ If a stage 3 study is required, original IDEAL guidelines on design and conduct of RCTs should be followed, including those on avoiding clinician bias affecting the consent process, and on using markers of the quality of delivery of the intervention to evaluate fidelity. The sub-framework highlights additional considerations including trial designs in global surgery. For example, cluster randomized trials may be more appropriate for public health interventions and stepped-wedge designs can also be considered.^17^ Employing adaptive trial designs by implementing protocol-driven preplanned interim evaluations that use prespecified updates or amendments of decision rules may also increase the efficiency and success of stage 3 studies.^18^ Master protocols and platform trial designs may be helpful for large trials across multiple countries as they can allow for the evaluation of multiple interventions within one stage 3 study, but the expertise and infrastructure required for these advanced trial designs may pose challenges. As with stage 2 studies, we recommend researchers include in-built qualitative process evaluations and consider phased or hybrid RCT-implementation evaluation designs to improve the interpretation of results in context, and the sustained uptake of innovation into future clinical practice.

#### IDEAL stage 4: engage with mixed-methods registries sooner

We recommend that registries be created and maintained as soon as the innovation is in general use to maximize the chance of detecting difficulties and minimizing the risk of missing safety concerns. These registries should preferably be a digital database to capture clinical safety concerns and efficiently report on these. These stage 4 studies can be started contemporaneously with earlier IDEAL stages during the innovation pathway where previously evaluated innovations are being adopted in a new context.

#### Pan-stage considerations and needs

A range of methodological considerations are considered relevant to all stages. These include the use of mixed methods to capture more information about how the innovation is being adopted within a given context, the adoption of a frugal innovation approach and the inclusion of a health economic evaluation where this is feasible. Respondents emphasized the need for access to funding and methodological expertise as the number one facilitating factor for high-quality evaluation. In many LMICs, there is also a need for collaboration between urban university hospitals with better resource and skills bases and the rural hospitals which serve most of the population. To develop local capacity, LMIC investigators need to be able to lead studies, but require mentoring and support.

## Discussion

The Global IDEAL Sub-Framework proposals increase the relevance and applicability of the IDEAL innovation evaluation pathway for surgical researchers working in low-resource environments. The recommendations provide valuable tools and considerations with the aim of improving evaluation of global surgical innovation.

Key strengths of this study include involving a wide range of participants from different backgrounds, geography and contexts which increases the generalizability of the recommendations. These recommendations were derived from and shaped by surgeons and researchers working in low-resource environments, ensuring the guidance is relevant and accessible. A further strength is aligning this new guidance to the original IDEAL Framework, an evidence-based pathway specifically for evaluating surgical innovation. The value added is the transformation of high-quality guidance into a more applicable and relevant tool for global surgical innovators.

The study also has important limitations. First, it was conducted in English only. This means we may have missed valuable information from non-English-speaking participants. Many countries were not represented despite efforts from the research team to ensure as wide a representation of contexts as possible. While the survey addressing barriers and facilitators had a range and number of respondents consistent with an adequate sample, the number of interviewees was not large enough to exclude the possibility of significant sampling error. The other IDEAL guidelines (IDEAL, IDEAL-D, DECIDE-AI and the IDEAL Robotics Colloquium) were developed using a multistakeholder expert conference to reach consensus, which gave them an important degree of authority and face validity. We intend to conduct this type of exercise and have therefore framed our findings as proposals rather than recommendations. Piloting of these proposals in practice could inform the deliberations of a future expert conference. This sub-framework will undergo iterative development with wider inclusion of specialties, geography, and contexts to improve the recommendations further.

Users of the Global IDEAL Sub-Framework who wish to conduct IDEAL studies in low-resource environments are therefore invited to visit the IDEAL Collaboration website (https://www.ideal-collaboration.net/projects/global-ideal/) for accompanying information and access to services including methodological support and innovation research dissemination. To encourage delivery of high-quality IDEAL Global evaluations, and to provide a future pathway to impact with reduced barriers, IDEAL Global is partnering with *BMJ Surgery, Interventions, & Health Technologies* to provide reduced fees or free publication for researchers from LMICs who submit studies using the IDEAL Global Framework.

## Data Availability

Data are available upon reasonable request. The results will be disseminated on social media platforms and reported back to funders.The data that support the findings of this study are available from the corresponding author, WSB, upon reasonable request.
