# Supplementary material for: No frugal innovation without frugal evaluation: the Global IDEAL Sub-Framework
Source: BMJ Surg Interv Health Technol. 2024 Jun 12;6(1):e000248. doi: 10.1136/bmjsit-2023-000248 (PMC11177672; doi:10.1136/bmjsit-2023-000248)
Supplement: Supplementary data [file bmjsit-2023-000248supp002.pdf]

< Return PREVIEW Innovation Evaluation in Global Surgery_v2 Skip: Next > 1 / 6

UNIVERSITY OF LEEDS

Evaluating Innovation and Technology in Global Surgery

0% complete

Page 1: Study information

Bolton WS, *et al. BMJ Surg Interv Health Technologies* 2024; 6:e000248. doi: 10.1136/bmjsit-2023-000248

Thank you for taking part in our survey. We are a group of researchers from the University of Leeds called the Global Health Research Group in Surgical Technologies (https://ghrgst.nihr.ac.uk).

This study aims to **develop a framework for the evaluation of innovation in global surgery**. The survey explores **barriers and facilitators** to evaluating innovation and technology in global surgery. In particular, how should we evaluate the adoption of an established technology in **a novel context or setting**?

The survey will take **no longer than 20 minutes** to complete. All the questions are in English. You do not have to complete the survey and your participation is entirely voluntary. You will not be asked for your name or email address as part of the survey itself, however once you complete the survey you **will have the opportunity to express your interest to be involved in a semi-structured interview about your experiences** with a member of the research team. This may be conducted online.

By completing this survey it implies you consent to the collection of this anonymous information on your experiences. Your data will be stored at the University of Leeds in accordance with GDPR regulations and a summary of anonymised data will be analysed and published in reports and articles. As we do not link any names or email addresses to your survey responses, you will be unable to withdraw your response once you complete the survey as we will not be able to link you personally to the data. If you begin the survey but change your mind about participating during the survey, you can simply close the window down and no data will be submitted.

If you have any questions about this study please email Dr William Bolton at the University of Leeds, w.s.bolton@leeds.ac.uk

If you would like further information on how the University uses personal data for research please see the Privacy Notice for Research.

This study has received ethical approval from the University of Leeds School of Medicine Research Ethics Committee MREC 18-102.

To start the survey, please click on the 'Next' button below.

Many thanks for your participation.

With best wishes,

Dr William Bolton on behalf of the NIHR Global Health Research Group in Surgical Technologies

Next >

Powered by online surveys | copyright | survey contact details | Report abuse

< Return PREVIEW Innovation Evaluation in Global Surgery_v2 Skip: < Previous Next > 2 / 6

UNIVERSITY OF LEEDS

Evaluating Innovation and Technology in Global Surgery

20% complete

Page 2: Some brief information about you...

1. Professional specialty (please check all that apply) *Required*

- ☐ Consultant Surgeon/Attending Physician
- ☐ Trainee/Resident Surgeon
- ☐ Surgical Associate/Surgical Officer
- ☐ Anaesthetist
- ☐ Obstetrician/Gynaecologist
- ☐ Researcher/Academic/Trialist/Methodologist
- ☐ Representative from industry, device manufacturer or related field
- ☐ Representative from regulatory related agency or related field
- ☐ Allied healthcare professional
- ☐ Patient/member of the public
- ☐ Other

2. What type of surgery best describes your practice? (tick all that apply) ✱ *Required*

- ☐ General
- ☐ Trauma & Orthopaedics
- ☐ Obstetrics & Gynaecology
- ☐ Paediatric
- ☐ Neurological
- ☐ Plastic and reconstructive
- ☐ Cardiac
- ☐ Urological
- ☐ Vascular
- ☐ ENT
- ☐ Ophthalmology
- ☐ Other
- ☐ N/A

3. Are you? ✱ *Required*

- ○ Male
- ○ Female
- ○ Other
- ○ Prefer not to say

3.a. If you selected Other, please specify:

[ ]

4. How old are you? ✱ *Required*

- ○ <30
- ○ 30-39
- ○ 40-49
- ○ 50-59
- ○ 60-69
- ○ 70+
- ○ Prefer not to say

5. What country(ies) are you currently working in with involvement in surgical care? (Please tick all that apply) ✱ *Required*

- ☐ Afghanistan
- ☐ Albania
- ☐ Algeria
- ☐ American Samoa
- ☐ Andorra
- ☐ Angola
- ☐ Antigua and Barbuda
- ☐ Argentina
- ☐ Armenia
- ☐ Aruba
- ☐ Australia
- ☐ Austria
- ☐ Azerbaijan
- ☐ Bahamas, The
- ☐ Bahrain
- ☐ Bangladesh
- ☐ Barbados
- ☐ Belarus
- ☐ Belgium

- ☐ Belize
- ☐ Benin
- ☐ Bermuda
- ☐ Bhutan
- ☐ Bolivia
- ☐ Bosnia and Herzegovina
- ☐ Botswana
- ☐ Brazil
- ☐ British Virgin Islands
- ☐ Brunei Darussalam
- ☐ Bulgaria
- ☐ Burkina Faso
- ☐ Burundi
- ☐ Cabo Verde
- ☐ Cambodia
- ☐ Cameroon
- ☐ Canada
- ☐ Cayman Islands
- ☐ Central African Republic
- ☐ Chad
- ☐ Channel Islands
- ☐ Chile
- ☐ China
- ☐ Colombia
- ☐ Comoros
- ☐ Congo, Dem. Rep.
- ☐ Congo, Rep.
- ☐ Costa Rica
- ☐ Côte d'Ivoire
- ☐ Croatia
- ☐ Cuba
- ☐ Curaçao

- [ ] Cyprus
- [ ] Czech Republic
- [ ] Denmark
- [ ] Djibouti
- [ ] Dominica
- [ ] Dominican Republic
- [ ] Ecuador
- [ ] Egypt, Arab Rep.
- [ ] El Salvador
- [ ] Equatorial Guinea
- [ ] Eritrea
- [ ] Estonia
- [ ] Eswatini
- [ ] Ethiopia
- [ ] Faroe Islands
- [ ] Fiji
- [ ] Finland
- [ ] France
- [ ] French Polynesia
- [ ] Gabon
- [ ] Gambia, The
- [ ] Georgia
- [ ] Germany
- [ ] Ghana
- [ ] Gibraltar
- [ ] Greece
- [ ] Greenland
- [ ] Grenada
- [ ] Guam
- [ ] Guatemala
- [ ] Guinea
- [ ] Guinea-Bissau

- ☐ Guyana
- ☐ Haiti
- ☐ Honduras
- ☐ Hong Kong SAR, China
- ☐ Hungary
- ☐ Iceland
- ☐ India
- ☐ Indonesia
- ☐ Iran, Islamic Rep.
- ☐ Iraq
- ☐ Ireland
- ☐ Isle of Man
- ☐ Israel
- ☐ Italy
- ☐ Jamaica
- ☐ Japan
- ☐ Jordan
- ☐ Kazakhstan
- ☐ Kenya
- ☐ Kiribati
- ☐ Korea, Dem. People's Rep.
- ☐ Korea, Rep.
- ☐ Kosovo
- ☐ Kuwait
- ☐ Kyrgyz Republic
- ☐ Lao PDR
- ☐ Latvia
- ☐ Lebanon
- ☐ Lesotho
- ☐ Liberia
- ☐ Libya
- ☐ Liechtenstein

- Lithuania
- Luxembourg
- Macao SAR, China
- Madagascar
- Malawi
- Malaysia
- Maldives
- Mali
- Malta
- Marshall Islands
- Mauritania
- Mauritius
- Mexico
- Micronesia, Fed. Sts.
- Moldova
- Monaco
- Mongolia
- Montenegro
- Morocco
- Mozambique
- Myanmar
- Namibia
- Nauru
- Nepal
- Netherlands
- New Caledonia
- New Zealand
- Nicaragua
- Niger
- Nigeria
- North Macedonia
- Northern Mariana Islands

- ☐ Norway
- ☐ Oman
- ☐ Pakistan
- ☐ Palau
- ☐ Panama
- ☐ Papua New Guinea
- ☐ Paraguay
- ☐ Peru
- ☐ Philippines
- ☐ Poland
- ☐ Portugal
- ☐ Puerto Rico
- ☐ Qatar
- ☐ Romania
- ☐ Russian Federation
- ☐ Rwanda
- ☐ Samoa
- ☐ San Marino
- ☐ São Tomé and Principe
- ☐ Saudi Arabia
- ☐ Senegal
- ☐ Serbia
- ☐ Seychelles
- ☐ Sierra Leone
- ☐ Singapore
- ☐ Sint Maarten (Dutch part)
- ☐ Slovak Republic
- ☐ Slovenia
- ☐ Solomon Islands
- ☐ Somalia
- ☐ South Africa
- ☐ South Sudan

- ☐ Spain
- ☐ Sri Lanka
- ☐ St. Kitts and Nevis
- ☐ St. Lucia
- ☐ St. Martin (French part)
- ☐ St. Vincent and the Grenadines
- ☐ Sudan
- ☐ Suriname
- ☐ Sweden
- ☐ Switzerland
- ☐ Syrian Arab Republic
- ☐ Tajikistan
- ☐ Tanzania
- ☐ Thailand
- ☐ Timor-Leste
- ☐ Togo
- ☐ Tonga
- ☐ Trinidad and Tobago
- ☐ Tunisia
- ☐ Turkey
- ☐ Turkmenistan
- ☐ Turks and Caicos Islands
- ☐ Tuvalu
- ☐ Uganda
- ☐ Ukraine
- ☐ United Arab Emirates
- ☐ United Kingdom
- ☐ United States
- ☐ Uruguay
- ☐ Uzbekistan
- ☐ Vanuatu
- ☐ Venezuela, RB

- ☐ Vietnam
- ☐ Virgin Islands (U.S.)
- ☐ West Bank and Gaza
- ☐ Yemen, Rep.
- ☐ Zambia
- ☐ Zimbabwe

6. How many operating theatres are in use in your facility? ✱ *Required*

- ○ 1-4
- ○ 5-9
- ○ 10+
- ○ N/A

7. Approximately how many beds does your hospital have in total? ✱ *Required*

- ○ less than 50
- ○ 50-99
- ○ 100-199
- ○ 200-499
- ○ 500-999
- ○ 1000+

8. Is your hospital ✱ *Required*

- ○ Public
- ○ Private
- ○ Mixed public and private
- ○ NGO/Charity

9. Does your hospital serve an area that is mostly * *Required*

- ○ Urban
- ○ Rural

< Previous

Next >

Powered by online surveys | copyright | survey contact details | Report abuse

< Return PREVIEW Innovation Evaluation in Global Surgery_v2 Skip: < Previous Next > 3 / 6

Evaluating Innovation and Technology in Global Surgery

40% complete

Page 3: Experience of evaluation

The Problem:

There is a balance between re-evaluating the intervention adequately versus the need to get the technology adopted in a timely manner. Currently, there is no clear pathway for implementing an established (in routine use) surgical intervention (technology or innovation) in a new/novel context or setting; for example, when an established technology from a high-income country (HIC) is to be implemented in a low and middle-income country (LMIC).

In this survey, we are asking key stakeholders like yourself to suggest barriers and facilitators to evaluating innovations and technology at different stages of development. We are also interested in what evidence you feel is required to inform the use and global adoption and in your specific healthcare setting setting.

Key Definitions:

Surgical procedure - a procedure where access is gained via an incision, natural orifice or percutaneous puncture or one that involves a device used inside the body.

Surgical technology/innovation - a device or equipment/technique used to perform the procedure.

10. What stage/s of the evaluation have you previously worked on? (tick all that apply) ✱ *Required*

- ☐ Pre-clinical (not in living humans)
- ☐ Clinical (with living humans)
- ☐ None

11. If you have experience in clinical evaluation, what stage of clinical evaluation did you work on? (tick all that apply) ✱ *Required*

- ☐ Small number of selected patients (n<10)
- ☐ Small number of selected patients (n>10)
- ☐ Prospective single group studies
- ☐ Single centre feasibility clinical trial with more than one study group (non-randomised)
- ☐ Multiple centre feasibility clinical trial with more than one study group (non-randomised)
- ☐ Single centre feasibility clinical trial with more than one study group (randomised)
- ☐ Multiple centre feasibility clinical trial with more than one study group (randomised)
- ☐ Single centre randomised controlled trial
- ☐ Multiple centre randomised controlled trial
- ☐ Long-term surveillance study (e.g. a registry)
- ☐ N/A

12. What classification was the surgical technology you worked on? (tick all that apply) ✱ *Required*

- ☐ Non-invasive (Either does not touch patient or in contact with intact skin/In contact with injured skin (mechanical barrier, compression, absorb exudates)
- ☐ Invasive (non-surgical): in body orifice or stoma
- ☐ Surgically invasive (transient/short term use): e.g. reusable surgical instruments, sutures/Supply/deliver energy or ionizing radiation/biological effect
- ☐ Surgically invasive (long-term and implantable devices):e.g. Implants/joint replacement/fixation devices.
- ☐ Active implantable devices: e.g. pacemakers
- ☐ Technology for the disinfection of medical devices
- ☐ None

< Previous Next >

Powered by online surveys | copyright | survey contact details | Report abuse

< Return PREVIEW Innovation Evaluation in Global Surgery_v2 Skip: < Previous Next > 4 / 6

UNIVERSITY OF LEEDS

Evaluating Innovation and Technology in Global Surgery

60% complete

Page 4: Barriers to innovation evaluation

Please rate your agreement with the following statements:

13. I have sufficient opportunity to evaluate surgical technology *Required*

- Strongly agree
- Agree
- Neutral
- Disagree
- Strongly disagree

14. I desire more opportunity to evaluate surgical technology *Required*

- Strongly agree
- Agree
- Neutral
- Disagree
- Strongly disagree

15. At present, conducting a prospective single group study is realistic at my facility/in my experience ✱ *Required*

- Strongly agree
- Agree
- Neutral
- Disagree
- Strongly disagree

This part of the survey uses a table of questions, view as separate questions instead?

15.a. The following are barriers to prospective single group studies in my setting (1 = NOT a barrier, 7 = significant barrier) ✱ *Required*

Please don't select more than 1 answer(s) per row.

Please select at least 8 answer(s).

	1	2	3	4	5	6	7
Lack of local collaborators	☐	☐	☐	☐	☐	☐	☐
Lack of international collaborators	☐	☐	☐	☐	☐	☐	☐
Lack of funding	☐	☐	☐	☐	☐	☐	☐
Previous bad experiences of developing/evaluating technology	☐	☐	☐	☐	☐	☐	☐
Lack of time	☐	☐	☐	☐	☐	☐	☐
Patient and public engagement	☐	☐	☐	☐	☐	☐	☐
Fear of lacking research expertise and skills in evaluation	☐	☐	☐	☐	☐	☐	☐
Lack of engagement from local policy makers	☐	☐	☐	☐	☐	☐	☐
Fear of lacking							

technical ability in development	☐	☐	☐	☐	☐	☐	☐
Lack of local industry engagement	☐	☐	☐	☐	☐	☐	☐
Lack of equipment and resources	☐	☐	☐	☐	☐	☐	☐
Lack of local human resources	☐	☐	☐	☐	☐	☐	☐
Challenges recruiting participants	☐	☐	☐	☐	☐	☐	☐

15.a.i. Other barriers: we may have missed some important barriers, please let us know based on your own experience

16. At present, conducting a single centre clinical trial with more than one study group (**non-randomised**) is realistic at my facility/in my experience *Required*

- Strongly agree
- Agree
- Neutral
- Disagree
- Strongly disagree

This part of the survey uses a table of questions, view as separate questions instead?

16.a. The following are barriers to single centre clinical trials with more than one study group (**non-randomised**) in my setting (1 = NOT a barrier, 7 = significant barrier)

Please don't select more than 1 answer(s) per row.

Please select at least 13 answer(s).

	1	2	3	4	5	6	7
Lack of local collaborators	☐	☐	☐	☐	☐	☐	☐

Lack of international collaborators	☐	☐	☐	☐	☐	☐	☐
Lack of funding	☐	☐	☐	☐	☐	☐	☐
Previous bad experiences of developing/evaluating technology	☐	☐	☐	☐	☐	☐	☐
Lack of time	☐	☐	☐	☐	☐	☐	☐
Patient and public engagement	☐	☐	☐	☐	☐	☐	☐
Fear of lacking research expertise and skills in evaluation	☐	☐	☐	☐	☐	☐	☐
Lack of engagement from local policy makers	☐	☐	☐	☐	☐	☐	☐
Fear of lacking technical ability in development	☐	☐	☐	☐	☐	☐	☐
Lack of local industry engagement	☐	☐	☐	☐	☐	☐	☐
Lack of equipment and resources	☐	☐	☐	☐	☐	☐	☐
Lack of local human resources	☐	☐	☐	☐	☐	☐	☐
Challenges recruiting participants	☐	☐	☐	☐	☐	☐	☐

16.a.i. Other barriers: we may have missed some important barriers, please let us know based on your own experience

17. At present, conducting a single centre clinical trial with more than one study group (**randomised controlled trial**) is realistic at my facility/in my experience *Required*

- Strongly agree
- Agree
- Neutral
- Disagree
- Strongly disagree

This part of the survey uses a table of questions, view as separate questions instead?

17.a. The following are barriers to single centre clinical trials with more than one study group (**randomised controlled trial**) in my setting (1 = NOT a barrier, 7 = significant barrier)

Please don't select more than 1 answer(s) per row.

Please select at least 13 answer(s).

	1	2	3	4	5	6	7
Lack of local collaborators	☐	☐	☐	☐	☐	☐	☐
Lack of international collaborators	☐	☐	☐	☐	☐	☐	☐
Lack of funding	☐	☐	☐	☐	☐	☐	☐
Previous bad experiences of developing/evaluating technology	☐	☐	☐	☐	☐	☐	☐
Lack of time	☐	☐	☐	☐	☐	☐	☐
Patient and public engagement	☐	☐	☐	☐	☐	☐	☐
Fear of lacking research expertise and skills in evaluation	☐	☐	☐	☐	☐	☐	☐
Lack of engagement from local policy makers	☐	☐	☐	☐	☐	☐	☐
Fear of lacking technical ability in development	☐	☐	☐	☐	☐	☐	☐

Lack of local industry engagement	☐	☐	☐	☐	☐	☐	☐
Lack of equipment and resources	☐	☐	☐	☐	☐	☐	☐
Lack of local human resources	☐	☐	☐	☐	☐	☐	☐
Challenges recruiting participants	☐	☐	☐	☐	☐	☐	☐

17.a.i. Other barriers: we may have missed some important barriers, please let us know based on your own experience

18. At present, conducting a multiple centre clinical trial with more than one study group (**randomised controlled trial**) is realistic at my facility/in my experience *Required*

- ○ Strongly agree
- ○ Agree
- ○ Neutral
- ○ Disagree
- ○ Strongly disagree

This part of the survey uses a table of questions, view as separate questions instead?

18.a. The following are barriers to multiple centre clinical trials with more than one study group (randomised controlled trial) in my setting (1 = NOT a barrier, 7 = significant barrier)

Please don't select more than 1 answer(s) per row.

Please select at least 13 answer(s).

	1	2	3	4	5	6	7
Lack of local collaborators	☐	☐	☐	☐	☐	☐	☐
Lack of international collaborators	☐	☐	☐	☐	☐	☐	☐

Lack of funding	☐	☐	☐	☐	☐	☐	☐
Previous bad experiences of developing/evaluating technology	☐	☐	☐	☐	☐	☐	☐
Lack of time	☐	☐	☐	☐	☐	☐	☐
Patient and public engagement	☐	☐	☐	☐	☐	☐	☐
Fear of lacking research expertise and skills in evaluation	☐	☐	☐	☐	☐	☐	☐
Lack of engagement from local policy makers	☐	☐	☐	☐	☐	☐	☐
Fear of lacking technical ability in development	☐	☐	☐	☐	☐	☐	☐
Lack of local industry engagement	☐	☐	☐	☐	☐	☐	☐
Lack of equipment and resources	☐	☐	☐	☐	☐	☐	☐
Lack of local human resources	☐	☐	☐	☐	☐	☐	☐
Challenges recruiting participants	☐	☐	☐	☐	☐	☐	☐

18.a.i. Other barriers: we may have missed some important barriers, please let us know based on your own experience

19. At present, conducting a long-term surveillance study (e.g. a registry) is realistic at my facility/in my experience *Required*

- ○ Strongly agree
- ○ Agree

- Neutral
- Disagree
- Strongly disagree

This part of the survey uses a table of questions, view as separate questions instead?

19.a. The following are barriers long-term surveillance studies (e.g. a registry) in my setting (1 = NOT a barrier, 7 = significant barrier)

Please don't select more than 1 answer(s) per row.

Please select at least 13 answer(s).

	1	2	3	4	5	6	7
Lack of local collaborators	☐	☐	☐	☐	☐	☐	☐
Lack of international collaborators	☐	☐	☐	☐	☐	☐	☐
Lack of funding	☐	☐	☐	☐	☐	☐	☐
Previous bad experiences of developing/evaluating technology	☐	☐	☐	☐	☐	☐	☐
Lack of time	☐	☐	☐	☐	☐	☐	☐
Patient and public engagement	☐	☐	☐	☐	☐	☐	☐
Fear of lacking research expertise and skills in evaluation	☐	☐	☐	☐	☐	☐	☐
Lack of engagement from local policy makers	☐	☐	☐	☐	☐	☐	☐
Fear of lacking technical ability in development	☐	☐	☐	☐	☐	☐	☐
Lack of local industry engagement	☐	☐	☐	☐	☐	☐	☐
Lack of equipment	☐	☐	☐	☐	☐	☐	☐

and resources							
Lack of local human resources	☐	☐	☐	☐	☐	☐	☐
Challenges recruiting participants	☐	☐	☐	☐	☐	☐	☐

19.a.i. Other barriers: we may have missed some important barriers, please let us know based on your own experience

[ ]

< Previous

Next >

Powered by online surveys | copyright | survey contact details | Report abuse

< Return PREVIEW Innovation Evaluation in Global Surgery_v2 Skip: < Previous Finish > 5 / 6

UNIVERSITY OF LEEDS

Evaluating Innovation and Technology in Global Surgery

80% complete

Page 5: Potential facilitators to innovation evaluation

20. How do you think we can overcome some of these barriers? In an ideal world (not limited by resources) what **3 to 6 things** do you think would most improve your opportunities and/or ability to engage with the evaluation of surgical technology and innovations? *Required*

21. Do you currently use, or are you aware of, any framework or guidelines that are designed to aid surgeons in the evaluation of surgical technology and innovations? (if none then leave blank) *Required*

22. An innovation/technology is evidence based and in routine use in a different setting to your own. In your opinion, what is the **minimum** evidence base required from studies conducted in your setting before it can be implemented and adopted? (Click 'None' if you think there are no additional studies required) ✱ *Required*

- ◯ None
- ◯ Prospective single group studies
- ◯ Single centre clinical trial with more than one study group (non-randomised)
- ◯ Single centre randomised controlled trial
- ◯ Multiple centre randomised controlled trial
- ◯ Long-term surveillance study (e.g. a registry)

23. Thinking about the current health facility where you work, on what basis is technology/innovation usually adopted into clinical practice? (Please tick all that apply) ✱ *Required*

- ☐ No evaluation
- ☐ Recommendation from colleagues
- ☐ Recommendation from industry representatives
- ☐ Based on clinical evaluation in countries other than your own
- ☐ Small clinical evaluation (none RCT) study in your own country
- ☐ RCT evidence in your own country

This part of the survey uses a table of questions, view as separate questions instead?

24. A technology/innovation is evidence based and in routine use in a setting other than your own. Before adopting it in your setting, how important is it to have evidence from evaluation with the following (1 = NOT important at all, 7 = EXTREMELY important) ✱ *Required*

Please don't select more than 1 answer(s) per row.

Please select at least 1 answer(s).

	1	2	3	4	5	6	7
Similar country income status	☐	☐	☐	☐	☐	☐	☐
Similar geographical area	☐	☐	☐	☐	☐	☐	☐
Similar healthcare system	☐	☐	☐	☐	☐	☐	☐
Similar healthcare facilities	☐	☐	☐	☐	☐	☐	☐
Similar patient population	☐	☐	☐	☐	☐	☐	☐

25. Apart from clinical trials, what other evaluation methodologies do you think could be useful to evaluate technology/innovation in global surgery? (Please tick all that apply) ✱ *Required*

- ☐ Implementation science
- ☐ Prospective registries
- ☐ Qualitative research
- ☐ Health systems research
- ☐ Process evaluations
- ☐ Health economics analysis
- ☐ None
- ☐ Other

< Previous

Finish ✔

Powered by online surveys | copyright | survey contact details | Report abuse

< Return PREVIEW Innovation Evaluation in Global Surgery_v2 < Previous 6 / 6

UNIVERSITY OF LEEDS

Evaluating Innovation and Technology in Global Surgery

100% complete

Final page

Thank you for completing the survey.

IMPORTANT: This section is not linked to your survey responses. We are conducting interviews online with participants who want to share more information about their experiences.

If you would like be approached about involvement in an interview about your experiences, please email Dr William Bolton at the University of Leeds, w.s.bolton@leeds.ac.uk

Powered by online surveys | copyright | survey contact details | Report abuse

Barriers and facilitators to surgical technology evaluation
Topic Guide

(Begin with standard introduction covering: introductions; process of conducting IDIs; consent)
Location:
Age:
Gender:

1. Did you complete the online survey?
2. Any feedback on the online survey content, format or length?
3. Please tell me about your current facility and clinical practice?
4. Please tell me more about your current country of work?

Previous experiences of technology evaluation

5. Please can you tell me about your experiences of evaluating technologies in global surgery?

   - What kinds of studies have you been involved in?
   - What were your roles in these studies?
   - What kinds of technologies have you been evaluating?
   - How did the study/s go?
   - Why did you want to get involved in these studies?

Perceived barriers of technology evaluation

6. What were the main barriers or challenges you faced when you did the study/s?

7. What are the challenges of conducing single group or single centre studies?

8. If you wanted to conduct larger or randomised studies, what would the main challenges be?

9. How could you do multicentre studies? What would the main challenges be there?

10. Is conducting a long term surveillance study (registry) feasible in your facility?

Perceived facilitators of technology evaluation

11. How do you think we can overcome some of the main barriers in your view?

12. How can we overcome the main barriers of delivering larger or randomised studies?

13. Are you aware of any guidelines or frameworks to help you design and deliver your studies? What would be useful in these?

14. If a technology is in current routine surgical practice in a high-income country (HIC), do you think this needs to be evaluated in full from small single group studies through to multicentre RCTs before it is adopted in LMICs?

15. How are new technologies routinely adopted in your facility?

16. Apart from trials, what other evaluation methodologies would be useful? Such as implementation science and qualitative research?

Overcoming some main challenges

17. How do you think we can improve human resource capacity?

18. How do you think we can improve funding for new technologies and clinical evaluation studies?

19. How do you think we can improve access to new technologies?

20. How can we persuade policy makers to adopt new technologies in global surgery?

Any questions for me?

Thanked.
