# Supplementary material for: No frugal innovation without frugal evaluation: the Global IDEAL Sub-Framework
Source: BMJ Surg Interv Health Technol. 2024 Jun 12;6(1):e000248. doi: 10.1136/bmjsit-2023-000248 (PMC11177672; doi:10.1136/bmjsit-2023-000248)

Appendix 1 – Pre-IDEAL: Stage Selection tool. This decision-making aid is designed to help the user identify the necessary existing evidence, how this relates to their context and then guides them to suitable study designs.

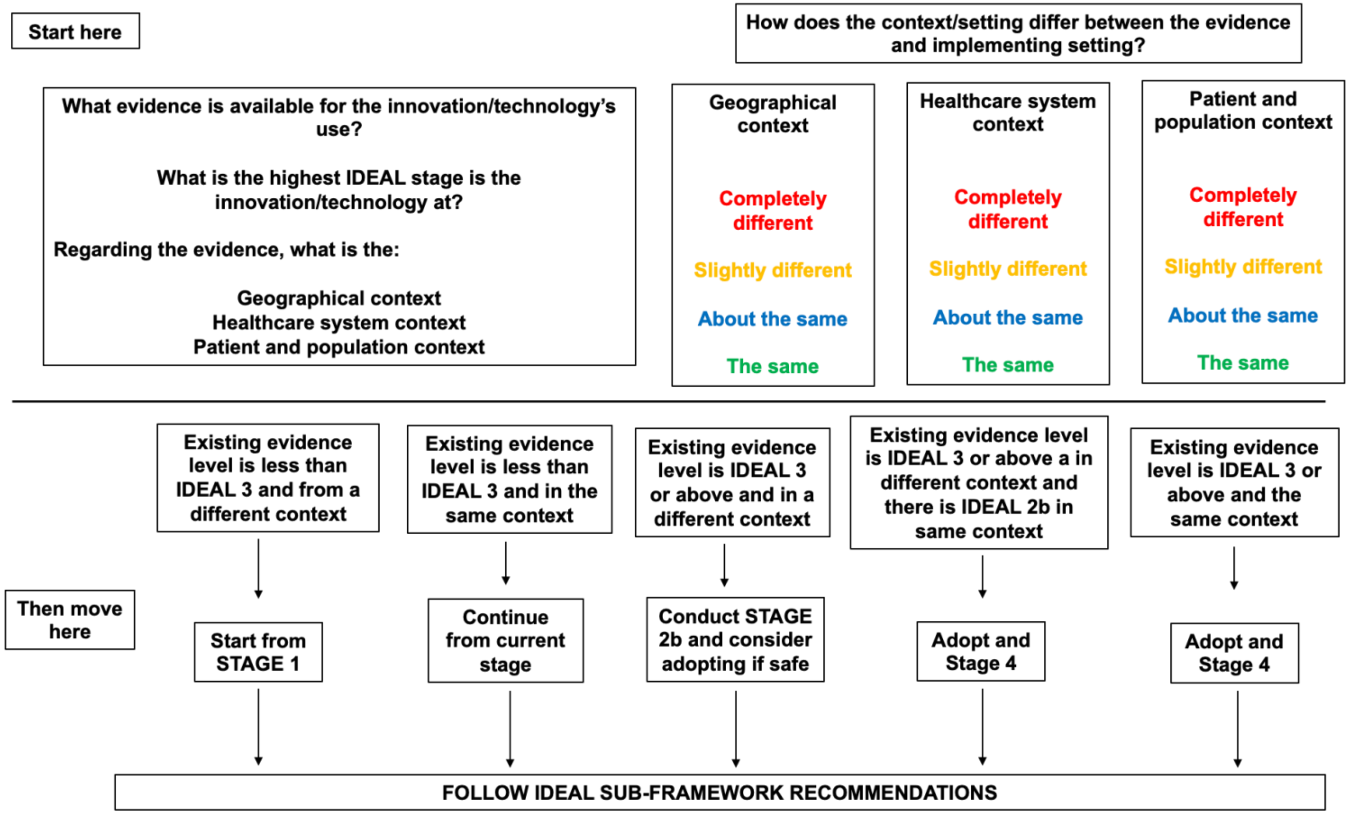

Supplement: Supplementary data [file bmjsit-2023-000248supp001.pdf]
